# Laser Depigmentation: A Case Report

**DOI:** 10.7759/cureus.64999

**Published:** 2024-07-20

**Authors:** Netal Kela, Shrishti Salian, Prasad Dhadse, Ruchita Patil, Sanehi Punse

**Affiliations:** 1 Department of Periodontics and Implantology, Sharad Pawar Dental College and Hospital, Datta Meghe Institute of Higher Education and Research, Wardha, IND

**Keywords:** esthetics, melanin, laser, hyperpigmentation, depigmentation

## Abstract

The pigmented lesions of the oral cavity may be endogenous or exogenous. Among the options for depigmenting these areas, laser therapy stands out for being a minimally invasive procedure. This study aims to report a clinical case of the gingival depigmentation technique using a high-power diode laser in the anterior maxillary region, for the ablation of the pigmented tissue to improve gingival aesthetics. The patient had an aesthetic complaint of the darkened aspect of the gingiva in the anterior maxillary portions. After local anesthesia, we started depigmentation with a high-power diode laser and performed ablation from the attached gingiva toward the free marginal gingiva. The patient returned after 30 and 180 days presenting healthy gingiva and absence of melanin repigmentation. Thus, we concluded that the diode laser was a good alternative for melanin depigmentation because it is a procedure with lower morbidity and satisfactory results.

## Introduction

The crucial components of a smile include the health and appearance of the gingiva [1]. Gingival hue varies among individuals and is thought to be associated with cutaneous pigmentation [2] According to a recent study, it was found that there is a strong association between skin tone and pigmentation [3]. It ranges in color from light to dark brown or black. Different races and geographical areas have different skin tones, textures, and colors [4]. The gingival epithelium thickness, the degree of keratinization, the amount and quantity of vasculature, and the pigments inside the epithelium are the crucial factors influencing gingival color [2].

Gingival hyperpigmentation is the term for when the normally pink gingival tissue becomes darker in certain situations. It is the condition where melanin deposition occurs in the suprabasal cell layers of the gingival epithelium. It is a multifactorial benign condition that causes esthetic concern to the person. Five main factors are responsible for the majority of pigmentations. These consist of carotene, oxyhemoglobin, decreased hemoglobin, melanin, and melanoid. Others are brought on by iron and bilirubin [5]. Melanin is a dark pigment that is not derived from hemoglobin, which is produced by melanocytes, located in the basal layer of the epithelium creating melanin, the most prevalent of the endogenous pigments. Gingival epithelium basal layer melanin staining occurs as a result of the granular melanin created by melanoblasts becoming tangled between epithelial cells. Racial or physiological gingival pigmentation is another name for gingival hyperpigmentation, which is believed to be a hereditary trait in certain age- or gender-specific populations. Additionally, gingival melanosis (also known as neurofibromatosis) is a common condition among ethnic groups with darker complexion [6].

The goal of gingival depigmentation is to treat gingiva that has become hyperpigmented with melanin. Several techniques have been employed, with varying degrees of success like the surgical scalpel approach, free gingival grafting, gingival abrasion, laser depigmentation, acellular dermal matrix allograft, cryosurgery, electrosurgery, and radiosurgery are examples of depigmentation treatments. Chemical methods include the use of substances such as phenols, ascorbic acid, and alcohol [7]. In recent times, melanin pigment-producing and -harboring cells have been ablated using lasers [8].

## Case presentation

**Figure 1:**
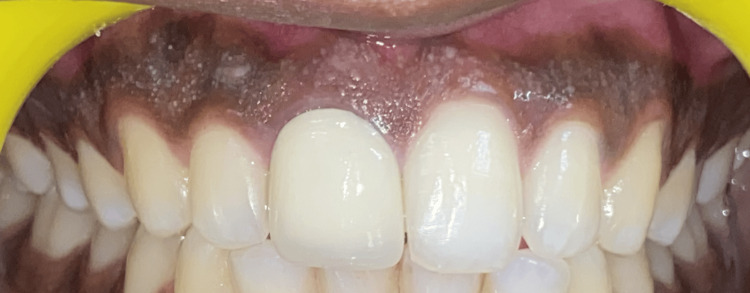
Pre-operative clinical view of the patient showing melanin pigmentation extending from 16-26.

A 19-year-old male patient came to the Department of Periodontics and Implantology with a chief complaint of poor aesthetics due to black gums. The past medical history was not significant. The past dental history suggested that it was a physiological melanin pigmentation that had existed since childhood. Figure 1 shows the pre-op clinical view. The patient maintained generally good and healthy dental hygiene.

**Figure 2:**
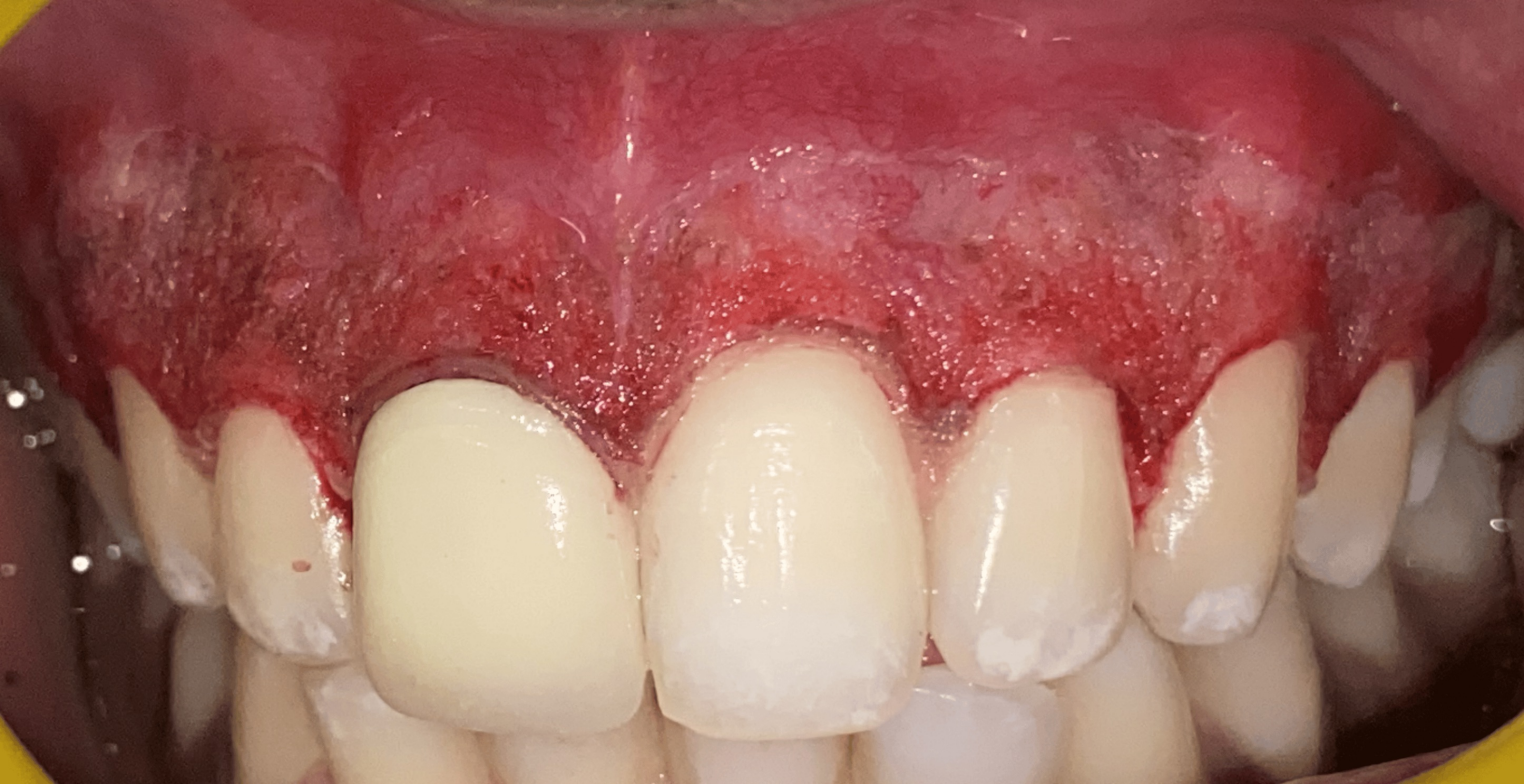
The surgical site immediately after depigmentation using a laser diode extending from 15-25 (according to the smile length).

After scaling during phase one therapy, the patient chose laser depigmentation from the available options for treatment. With the consent of the patient, local anesthesia (local infiltration) was administered. For depigmentation, a stent made of cellophane sheet was fabricated and a laser diode was employed, spanning the maxillary arch from the right second premolar to the left second premolar. Depigmentation was done in the horizontal direction, corresponding to the root surfaces, on the pigmented gingiva, using the laser point in contact mode to prevent overheating. Figure 2 shows the site of interest immediately after laser depigmentation. After that, gauze soaked in saline was used to cleanse the depigmented area.

**Figure 3:**
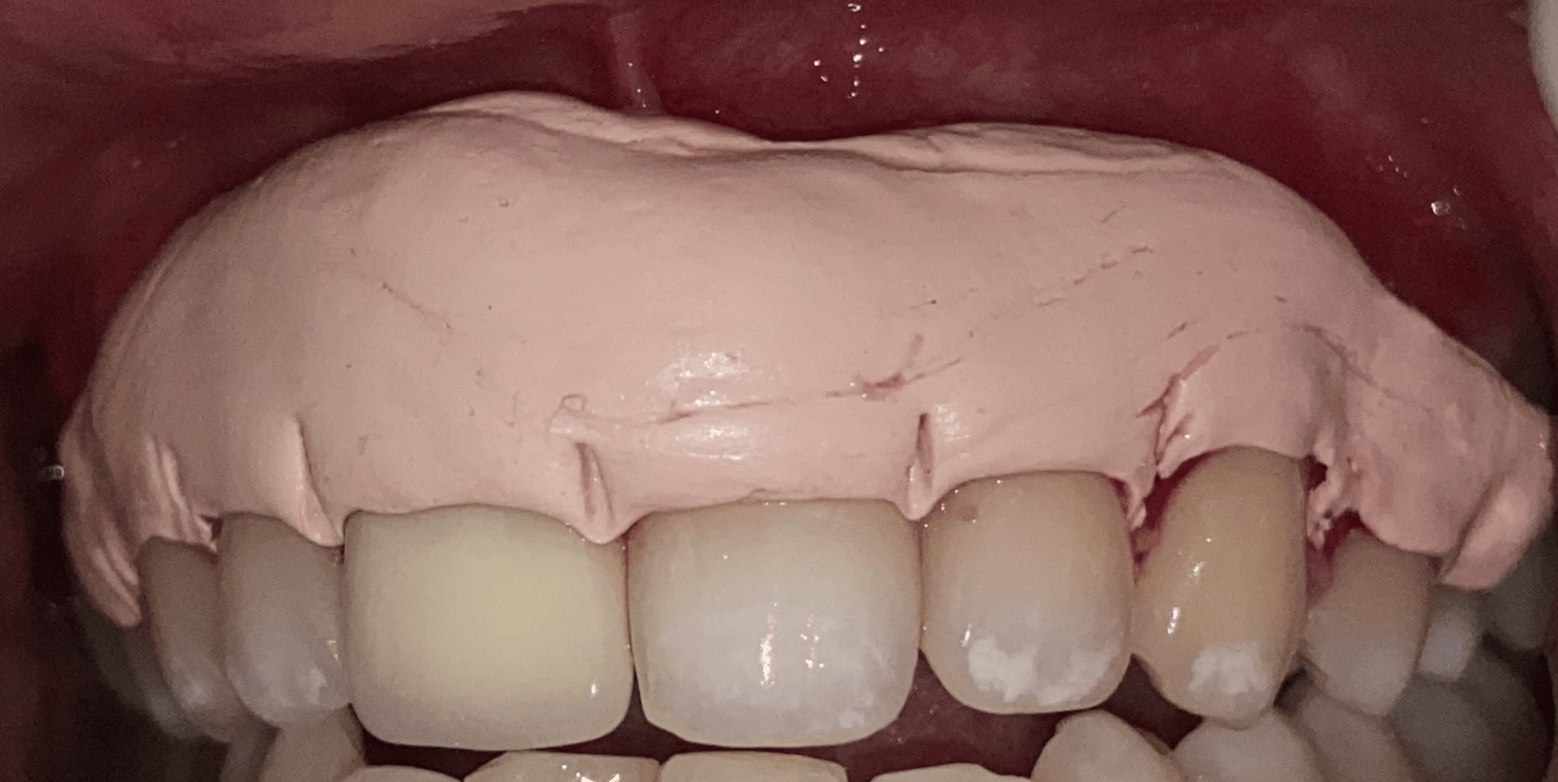
Periodontal pack placement extending from 15-25.

**Figure 4:**
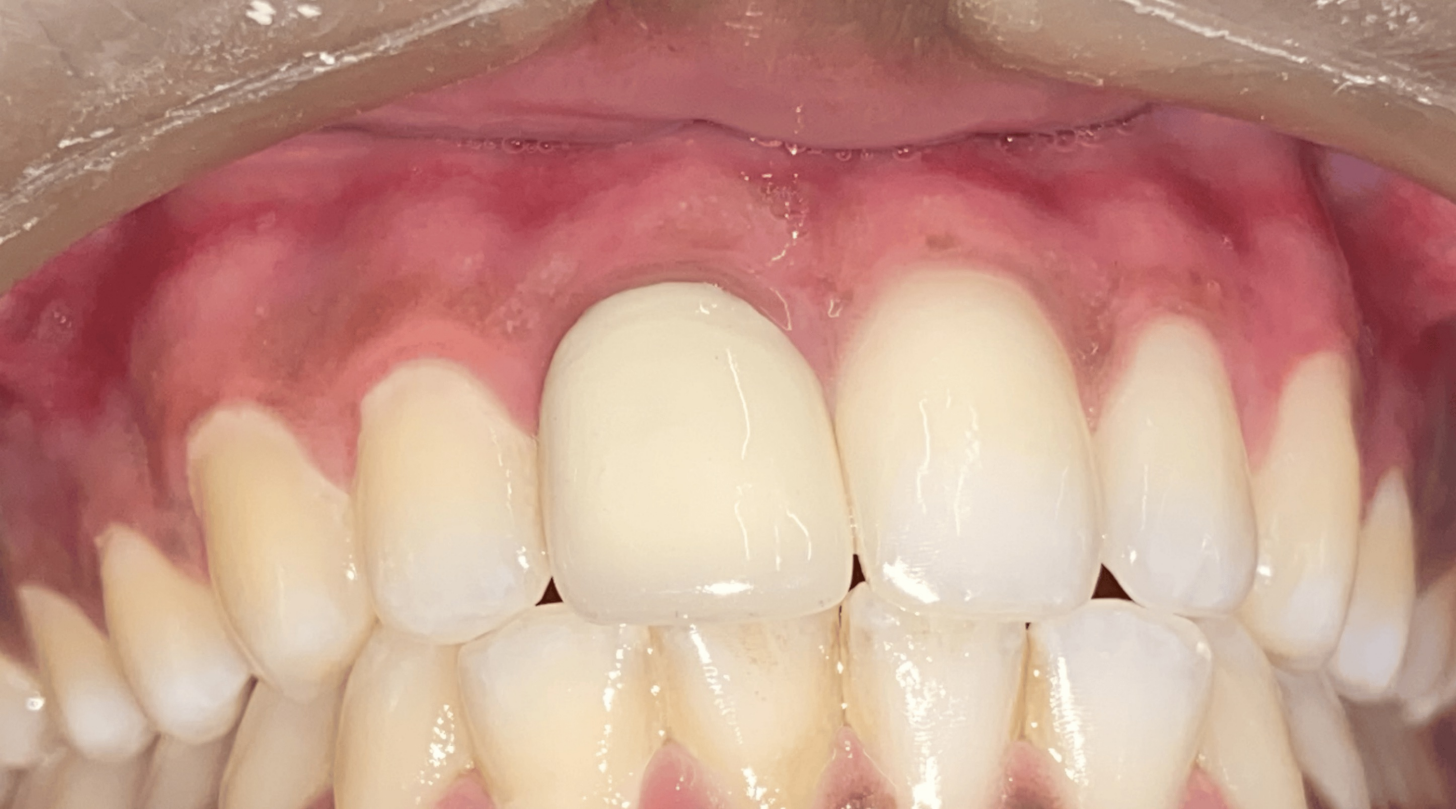
Three-month post-operative view shows reduced melanin pigmentation.

Throughout the procedure, the patient was at peace, and there was no bleeding. The patient received post-operative instructions and a periodontal pack was placed (Figure 3). After a week, the patient was recalled for a follow-up assessment, with no complaint of pain in the relevant location. The periodontal pack was taken out. Both arches healed without any complications. A three-month follow-up revealed that the gingiva was healthy, pink, and firm, thus everything appeared normal (Figure 4). There was no recorded recurrence, and the patient was extremely happy with the outcome.

## Discussion

People of all ethnicities are affected by oral pigmentation. The differences between males and females are not so great [9]. Conversely, the pigmentation of the oral epithelium differs in distribution and strength. It varies not just between races but also inside a person's mouth cavity and among members of the same race. Physiologic pigmentation is most likely influenced by inheritance; however, mechanical, chemical, and physical variables also regulate the degree of pigmentation.

In the skin, melanin carries out vital, necessary, and crucial tasks like acting as an antioxidant, offering photoprotection against UV radiation, and even having the ability to bind drugs, like nicotine in smokers. Most noteworthy, though, in the context of depigmentation, is the fact that lasers offer a painless, minimally intrusive, and efficient substitute for the more traditional and conventional methods of depigmentation. The best treatment for gingival darkening is laser therapy [10]. “Yttrium aluminum garnet (Nd: YAG, 1,064 nm), carbon dioxide (CO_2_, 10,600 nm), and diode (980 nm)” when it comes to gingival depigmentation, these lasers are the most frequently employed type. Lasers make surgical sites readily visible, and they also reduce the risk of post-operative complications like pain, hemorrhage, edema, infection, and sluggish wound healing [11]. The interdental papilla is an easy-to-access area with a lower relapse incidence, making it a safe and dependable treatment option.

Usually, single laser treatment is enough to remove the pigmented spots; no periodontal dressing is needed. The advantages of this include ease of handling, immediate therapies, hemostasis, and decontamination and sterilization outcomes. However, this method requires pricey, complex equipment, which is not widely available and raises the cost of the treatment [12]. While the wavelength, thermal result, and tissue properties of each laser dictate the absorption rate, the emission parameters of specific laser systems can affect the thermal effect on soft tissue. Even though hemoglobin and melanin absorb the majority of the 980 nm diode laser's energy, the high energy density level required for photothermal soft tissue ablation makes it difficult to utilize such a diode therapeutically without seriously damaging adjacent tissue. Consequently, low power levels (0.8 W) must be used to start the diode laser tip to increase its temperature. Tissue excision can be accomplished by taking advantage of the heating effect making contact with the target tissue by the laser point.

Diode lasers are located in the melanin absorption spectrum and have a deeper penetration. The heating effect of the diode laser causes restriction of blood vessels encircling the tissue, which could delay the migration of melanocytes. Furthermore, the diode laser may be absorbed by melanophages or melanophores, the pigment-containing cells, that may have become trapped in the lamina propria. Additionally, it has specific effects on cells that lower their activity. Comparing diode lasers to other solid-state and gas lasers, they are quieter, lighter, and more portable due to their high electrical to optical efficiency. The laser is an effective soft tissue surgical tool, suitable for soft tissue curettage or sulcular debridement, as well as for cutting and coagulating gingiva and oral mucosa because the diode fails to communicate with dental hard tissues at lower power values.

Hassan et al. (2022) provided a comparison of the efficacy of laser and scalpel procedures for treating gingival hyperpigmentation. A 23-year-old female patient had diode laser treatment on her left side and scalpel treatment on her right. The authors concluded that there was no recurrence with any of the therapies they examined and that both techniques had similar healing outcomes. The charred layer stopped the bleeding, while the scalpel method left a bleeding region that needed to be examined after the procedure [13]. In a case study by Elemek, depigmentation treatment with an ‘810 nm diode laser’ was carried out in two female patients. The patients were contacted again at weeks one, four, and twelve to evaluate the rate of healing and recurrence. This study shows that depigmentation using an 810 nm diode laser is aesthetically pleasing and comfortable for patients [14].

## Conclusions

In every case, depigmentation is a voluntary cosmetic operation motivated by the patient's desire for a gorgeous smile. For the removal of unsightly gingival melanin pigmentation, the diode laser is a safer treatment option. It also provides individuals with gingival hyperpigmentation with acceptable final aesthetics and the least amount of discomfort possible.
